# V*ß*Gene Family Usage in Spontaneous Lymphomas of AKR Mice: Evidence for Defective Clonal Deletion

**DOI:** 10.1155/1992/91527

**Published:** 1992

**Authors:** Cees de Heer, Bernard de Geus, Henk-Jan Schuurma, Henk Van Loveren, Jan Rozing

**Affiliations:** 1 Laboratory for Pathology National Institute of Public Health and Environmental Protection Bilthoven The Netherlands; 2 Division of Histochemistry and Electron Microscopy Departments of Pathology and Internal Medicine University Hospital Utrecht The Netherlands; 3 Department of Immunotoxicology Laboratory for Pathology National Institute of Public Health and Environmental Protection P.O. Box 1 Bilthoven 3720 BA The Netherlands; 4 Department of Immunology TNO Institute for Aging and Vascular Research Leiden The Netherlands

**Keywords:** Lymphomagenesis, AKR, thymus, selection, V*ß*

## Abstract

T-cell receptor (TCR) *ß*-chain usage and expression of the CD3, CD4, and CD8 differentiation antigens were analyzed in 14 spontaneous AKR lymphomas. Lymphoma cells massively infiltrated and/or proliferated in the organs analyzed (thymus, spleen, and mesenteric lymph nodes), giving rise to a loss of organ structure. One lymphoma occurred only in the thymus, and failed to express CD3, CD4, and CD8. All other lymphomas expressed the CD3/TCR complex. With respect to CD4 and CD8 expression, the lymphomas were either double-negative (DN), double-positive (DP), or single-positive (SP). The frequency of DP (CD4^+^8^+^) lymphomas was low compared to the frequency of DP thymocytes in a normal AKR thymus. A substantial heterogeneity was seen in the intensity of CD4 and CD8 expression among various lymphomas, which was independent of the level of CD3 expression. Considering TCR V *ß* gene family usage, 2 out of 14 lymphomas expressed V *ß*6. Normally, V *ß*6^+^ thymocytes are deleted from the thymocyte pool at the immature DP stage of T-cell development in AKR mice. These data support the hypothesis that the lymphocytes in the immature DP stage of T-cell development are susceptible to the induction of AKR lymphomagenesis. The presence of  V *ß*6^+^ lymphoma cells indicates that the lymphomagenesis is accompanied by a defective clonal deletion of cells expressing a possible autoreactive TCR.


*Developmental Immunology*, 1992, Vol. 2, pp. 95–101
Reprints available directly from the publisher
Photocopying permitted by license only

© 1992 Harwood Academic Publishers GmbH
Printed in the United Kingdom

### Vβ Gene Family Usage in Spontaneous Lymphomas of AKR Mice: Evidence for Defective Clonal Deletion

CEES DE HEER,*†‡§ BERNARD DE GEUS,§ HENK-JAN SCHUURMAN,†‡ HENK VAN LOVEREN,† and JAN ROZING§

†*Laboratory for Pathology, National Institute of Public Health and Environmental Protection, Bilthoven, The Netherlands*
‡*Division of Histochemistry and Electron Microscopy, Departments of Pathology and Internal Medicine, University Hospital, Utrecht, The Netherlands*
§*Department of Immunology, TNO Institute for Aging and Vascular Research, Leiden, The Netherlands*

T-cell receptor (TCR) β-chain usage and expression of the CD3, CD4, and CD8 differentiation antigens were analyzed in 14 spontaneous AKR lymphomas. Lymphoma cells massively infiltrated and/or proliferated in the organs analyzed (thymus, spleen, and mesenteric lymph nodes), giving rise to a loss of organ structure. One lymphoma occurred only in the thymus, and failed to express CD3, CD4, and CD8. All other lymphomas expressed the CD3/TCR complex. With respect to CD4 and CD8 expression, the lymphomas were either double-negative (DN), double-positive (DP), or single-positive (SP). The frequency of DP ($CD4^+8^+$) lymphomas was low compared to the frequency of DP thymocytes in a normal AKR thymus. A substantial heterogeneity was seen in the intensity of CD4 and CD8 expression among various lymphomas, which was independent of the level of CD3 expression. Considering TCR Vβ gene family usage, 2 out of 14 lymphomas expressed Vβ6. Normally, $V\beta6^+$ thymocytes are deleted from the thymocyte pool at the immature DP stage of T-cell development in AKR mice. These data support the hypothesis that the lymphocytes in the immature DP stage of T-cell development are susceptible to the induction of AKR lymphomagenesis. The presence of $V\beta6^+$ lymphoma cells indicates that the lymphomagenesis is accompanied by a defective clonal deletion of cells expressing a possible autoreactive TCR.

KEYWORDS: Lymphomagenesis, AKR, thymus, selection, Vβ.

### INTRODUCTION

AKR mice have a high incidence of spontaneous (thymic) lymphomas that develop after the appearance of a functional polytropic retrovirus. The thymus appears to be the most common site of this neoplastic process, and is in some cases the only organ involved. The crucial role of the thymus in the lymphomagenesis is also shown because the incidence of lymphomas is decreased after thymectomy in young healthy mice (1–2 months old), but not after splenectomy. Both the stationary (epithelial) and bone marrow-derived (macrophage, dendritic) stromal components of the thymic microenvironment have been claimed to contribute to the lymphomagenesis (Tempelis, 1987; Kim et al., 1991). Thymic macrophages in the corticomedullary region are the first cells in which detectable levels of functional polytropic retrovirus are found (Kim et al., 1991). On the other hand, also the radioresistant thymic epithelial cells are able to amplify or suppress expression of AKR retroviruses and the prelymphomic phenotypical changes (Tempelis, 1987). The developmental stage, in which the differentiating thymocytes become susceptible to the induction of the lymphomagenesis, is nevertheless not known.

The epithelial and bone marrow-derived cells in the thymus also play an important role in shaping the repertoire of mature peripheral T cells. Thymocyte maturation involves positive and negative selection processes that generate mature T cells that are both self-tolerant as well as able to recognize antigen in the context of self-

*Corresponding author. Present address: Department of Immunotoxicology, Laboratory for Pathology, National Institute of Public Health and Environmental Protection, P.O. Box 1, 3720 BA Bilthoven, The Netherlands.

major histocompatibility complex (MHC) determinants (Ramsdell and Fowlkes, 1990). Interactions between developing T cells and stromal elements in the thymus are essential for these processes. The CD3/T-cell antigen receptor complex, MHC determinants, and the CD4 or CD8 differentiation antigens are involved in these interactions (Ramsdell and Fowlkes, 1990).

AKR mice have the minor lymphocyte stimulatory locus-1$^a$ (Mls-1$^a$) phenotype. Mls-1$^a$ was considered a minor histocompatibility antigen, but has recently been identified as an endogenous retroviral antigen (Frankel et al., 1991). Mls-1$^a$ mice are deleting their Mls-1$^a$ reactive T lymphocytes during intrathymic maturation (Kappler et al., 1988; MacDonald et al., 1988a; Happ et al., 1989), that is, T cells expressing the V$\beta$6, V$\beta$8.1 or V$\beta$9 variable gene segments of the $\beta$ chain of the T-cell receptor (TCR). The response to Mls-1$^a$ is restricted to MHC class II, and the CD4$^+$ T-cell subset is the presumed autoreactive population. It was shown that V$\beta$6$^+$CD8$^+$ T lymphocytes alone do not give an anti-Mls-1$^a$ proliferative response *in vitro,* and need the help of CD4$^+$ T cells (MacDonald et al., 1988a; Webb and Sprent, 1990). However, all T cells expressing these TCR are expected to be deleted from the thymocyte pool of the AKR thymus (MacDonald et al., 1988a) at the immature double-positive (DP) stage of T-cell development (Blackman et al., 1990b; Matsuzaki et al., 1990; Ramsdell and Fowlkes, 1990). This results in the absence of V$\beta$6, V$\beta$8.1, and V$\beta$9 expressing CD4$^+$ and CD8$^+$ single-positive (SP) T cells in the mature thymocyte and peripheral T-cell populations of normal AKR mice (MacDonald et al., 1988a; Webb and Sprent, 1990). Rearrangement of the V$\beta$6 gene has been described in one AKR lymphoma (Lee and Davis, 1988), but it has not been documented whether this rearrangement was functional and resulted in the expression of V$\beta$6 on the cell surface.

We analyzed 14 naturally occurring AKR lymphomas using monoclonal antibodies (mAB) to the CD3, CD4, and CD8 differentiation antigens, and to TCR $\beta$-chain variable region segments V$\beta$3, V$\beta$6, and V$\beta$8.1/2. This latter mAB recognizes an epitope shared by V$\beta$8.1 and V$\beta$8.2, but not other members of the V$\beta$8 family. Our data indicate that the immature DP (CD4$^+$8$^+$) thymocyte is the most likely target for AKR lymphomagenesis. The lymphomagenesis is accompanied by defects in the clonal deletion of lymphoma cells that express autoreactive TCR.

### RESULTS

The greatly enlarged lymphoid organs (thymus, spleen, mesenteric lymph nodes) of the lymphomic AKR mice showed lymphoma cells in thymus, spleen, and lymph nodes in all but one of the AKR mice. In one case (case 11), the lymphoma was present in the thymus only. In the lymphoid tissues of some of the lymphoma-bearing animals, remnants of the preexistent organ structure were observed. In the majority of cases, there was a massive infiltration and/or proliferation of lymphoma cells with a loss of the normal structure of the lymphoid organs.

In immunohistochemistry for CD3/TCR complex, CD4, and CD8, all but one of the lymphomas expressed the CD3/TCR complex indicating the T-cell lineage origin of the lymphomas. Staining for CD4 and CD8 enabled identification of lymphomas with a double-negative (DN), DP, and SP phenotype (Table 1). The frequencies of these phenotypes differ from that in the normal AKR thymus, with a shift to the more mature SP phenotypes (Table 1). The intensity of CD4 and CD8 expression varied widely between individual lymphomas, independently of the intensity of

TABLE 1
CD3/TCR and CD4/CD8 Expression on Thymic Lymphomas in AKR Mice[a]

| Case | CD4 | CD8 | CD3/T-cell receptor | |
|---|---|---|---|---|
| 11 | – | – | – | |
| 1b[b] | – | – | + | |
| 2 | – | – | + | |
| 5 | – | – | + | |
| 13 | – | – | $+^{low}$ | |
| 4a[b] | $+^{high}$ | – | + | V$\beta$6 |
| 4b[b] | $+^{low}$ | – | $+^{high}$ | V$\beta$8.1/2[c] |
| 6 | + | – | + | |
| 7 | $+^{low}$ | – | + | V$\beta$8.1/2[c] |
| 14 | + | – | + | V$\beta$6 |
| 15 | $+^{low}$ | – | + | |
| 1a[b] | – | + | + | |
| 9 | – | + | + | |
| 10 | – | + | + | V$\beta$3 |
| 8 | + | $+^{low}$ | $+^{low}$ | |
| 12 | $+^{high}$ | + | + | V$\beta$8.1/2[c] |

[a]"High" and "low" indicate expression intensities that are significantly higher or lower than the average intensity.

[b]Cases 1 and 4 were of biclonal origin; the clones were designated as a and b. These clones were not overlapping, i.e., cells expressing V$\beta$6 and V$\beta$8.1/2 were localized at different positions in immunohistology (Fig. 1).

[c]All KJ16-positive AKR lymphomas expressed the V$\beta$8.2 gene segment as shown by polymerase chain-reaction analysis (Fig. 2).

CD3/TCR-complex expression (Table 1). Staining with the anti-CD3 mAB YCD3-1 revealed a rare thymic phenotype in four of the DN lymphomas; these four cases expressed CD3 at a readily detectable intensity. The one CD3-negative lymphoma case corresponded to a presumed immature triple-negative stage ($CD3^-4^-8^-$) of T-cell development.

Further examination of the primary lymphomas with mABs to Vβ gene family products of the TCR revealed a large difference in Vβ usage between the lymphoma cases and normal AKR thymocytes or peripheral T lymphocytes. Five out of 14 lymphomas were positive for Vβ3, Vβ6, or Vβ8.1/2 (Table 1), whereas the other lymphomas were negative for these families. One lymphoma (case 10) expressed Vβ3 and two lymphomas expressed Vβ6 (case 4a, Fig. 1A; and case 14). Three lymphomas expressed Vβ8.1 and/or Vβ8.2 (case 4b, Fig. 1B; and cases 7 and 12). By using polymerase chain-reaction technology, all three cases were shown to express the Vβ8.2 gene segment (Fig. 2), and not Vβ8.1. Two of the lymphomas appeared to be of biclonal origin (cases 1 and 4). For case 4, this was illustrated by family-specific antibodies, that is, one identifying the clone bearing Vβ6 and one identifying the clone bearing Vβ8.1/2. These two clones differed also in intensity of CD4 expression and expression of the CD3/TCR complex, and in tissue localization (Fig. 1). This rules out the possibility that a failure in allelic exclusion occurred in this lymphoma leading to the expression of two TCR β chains on one cell.

In all lymphomas, varying numbers of CD4 and CD8 SP cells occurred with a phenotype different from that of the lymphoma, presumably representing preexistent T cells. These CD4 and CD8 SP populations appeared to be present in a normal CD4/CD8 ratio, compared to the mature thymocyte and peripheral T-cell population.

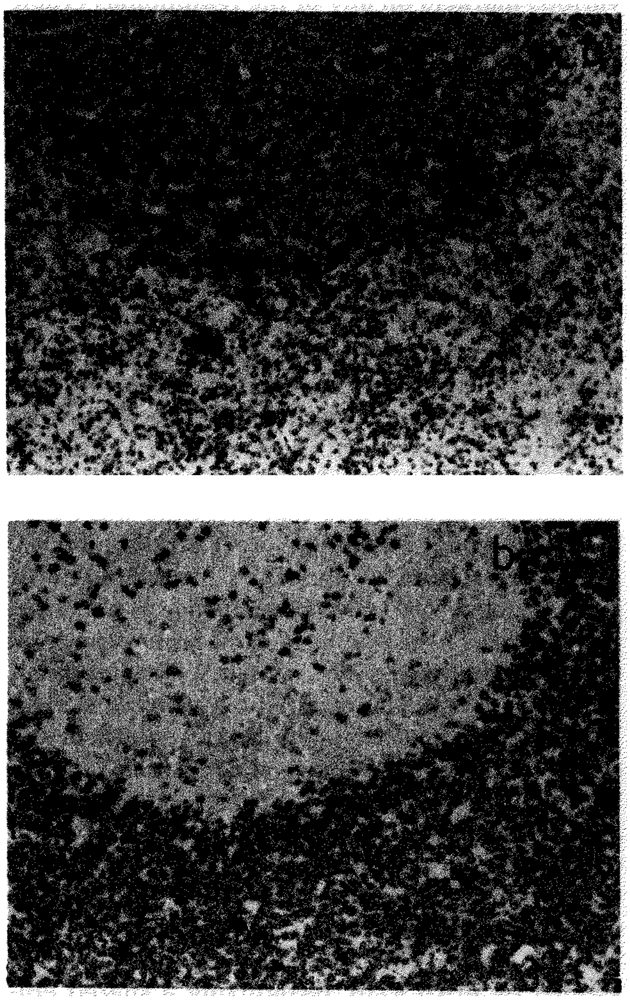

FIGURE 1. Immunoperoxidase staining of serial cryostat sections of the lymphomic spleen of case 4 with (a) mAB 44-22-1 (anti-Vβ6) and (b) mAB KJ16 (anti-Vβ8.1/2). Note the different localization of the two clones, one bearing Vβ6 and one bearing Vβ8.1/2.

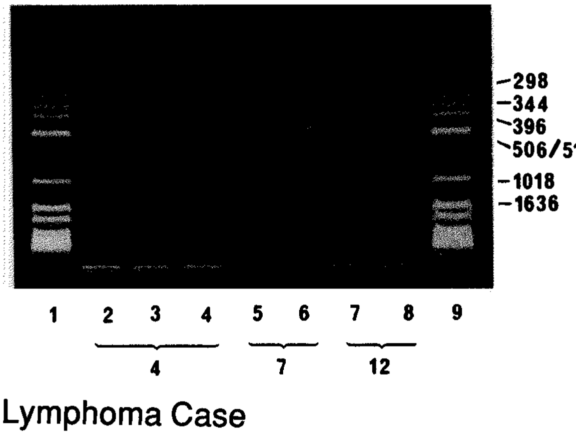

FIGURE 2. T-cell receptor β-chain PCR products from AKR lymphoma genomic DNA. Lanes 1 and 9: 1 kb DNA ladder (Gibco/BRL, Bethesda, MD); lane 2: case 4, Vβ6; lane 3: case 4, Vβ8.1; lane 4: case 4, Vβ8.2; lane 5: case 7, Vβ8.1; lane 6: case 7, Vβ8.2; lane 7: case 12, Vβ8.1; and lane 8: case 12, Vβ8.2.

### DISCUSSION

Of the AKR mice investigated, the predominant presence of lymphoma cells was shown in all lymphoid organs. This was accompanied by a loss of organ structure and presence of only remnants of the preexistent stroma. One case (case 11) was exceptional, as in this case, the lymphoma was only restricted to the thymus. Strikingly, this lymphoma represented the most immature phenotype of all cases studied, suggesting an inability of this lymphoma to migrate to the periphery possibly due to its arrest in an immature stage of development. The T-cell lineage origin of the lymphomas was evident from the expression of the CD3/TCR complex, in all but one (case 11) of the cases.

CD4 and CD8 phenotyping of the lymphomas revealed DN ($CD4^-8^-$), DP ($CD4^+8^+$), and SP ($CD4^+8^-$ or $CD4^-8^+$) lymphomas. The percentages of these different phenotypes (30%, $CD4^-8^-$; 13%, $CD4^+8^+$; 38%, $CD4^+8^-$; and 19%, $CD4^-8^+$) did not correspond to the percentages of these populations in the normal AKR thymus, that is, 4%, 81%, 10%, and 5%, respectively (Fig. 3). This is especially true for the DP phenotype.

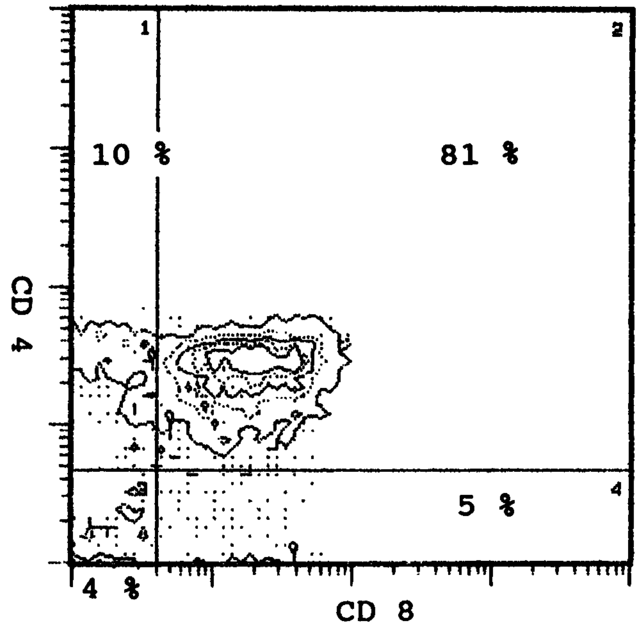

FIGURE 3. Expression of CD4 and CD8 differentiation antigens on normal AKR thymocytes. The percentages given indicate the percentage of gated cells falling within the quadrant of the two-dimensional contour plot.

The present phenotype distribution among the lymphomas deviates from the findings of Richie et al. (1988), who found a more frequent occurrence of immature phenotypes ($CD4^-8^-$ and $CD4^+8^+$) in a series of 12 naturally occurring AKR lymphomas. Combined with our data, there is no prevalent phenotype of the AKR lymphomas. This may suggest that there is no distinct phenotype that is particularly susceptible for lymphomagenesis. However, other data in fact do indicate a preferential phenotype of AKR lymphomas. Within the different populations based on CD4/CD8 phenotype, there was a large heterogeneity in the intensity of expression of the CD3, CD4, and CD8 antigens. There was no common pattern in the expression levels of the various markers on any of the lymphomas (Table 1). This variability in CD3, CD4, and CD8 expression suggests a random loss of membrane antigens. Such a loss of cell membrane markers is well-known for T-cell neoplasms (Schuurman et al., 1991). It is therefore tempting to speculate that most of the SP lymphomas are derived from a DP lymphomic progenitor, and hence the number of DP lymphomas in this study may be an underestimate of the real value. The expression of CD3 on the DN lymphoma cells is in line with this explanation. These observations point to the possibility that the immature DP stage of T-cell development is the target for the induction of the lymphomagenesis, with subsequent antigenic loss and development of SP, DN, or even $CD3^-$ cases of lymphoma, and that therefore there may be a preferential phenotype of AKR lymphomas.

The expression of the TCR $\beta$-chain variable gene segment V$\beta$6 on the cell surface of two lymphoma cases also points to lymphomagenesis at the immature DP stage of T-cell development. Normally, in AKR mice, thymocytes expressing a V$\beta$6 TCR are deleted at that stage from the thymocyte pool in AKR mice (MacDonald et al., 1988b). Two out of 14 lymphomas tested (cases 4a and 14) expressed a V$\beta6^+$ TCR. Both were of the $CD4^+8^-$ phenotype (Table 1), which suggests that these lymphomas belong to the "forbidden" $CD4^+$ autoreactive population (MacDonald et al., 1988a). Three lymphomas expressed a TCR stained by mAB KJ16, recognizing a shared epitope on V$\beta$8.1 and V$\beta$8.2 (V$\beta$8.1 being "forbidden"). In genomic DNA analysis, all three $KJ16^+$ lymphomas expressed the V$\beta$8.2 TCR (Fig. 2), which is not autoreactive. Therefore, only the two Vβ6 expressing lymphomas are potentially autoreactive.

During T-cell development, positive selection precedes negative selection (Blackman et al., 1990b; Ramsdell and Fowlkes, 1990). The presence of the $V\beta6^{+}CD4^{+}$ lymphomas indicates that the lymphomagenesis occurs after or during positive selection, but before negative selection. This implies that interaction between precursors of lymphoma cells and the thymic microenvironment was not effective in creating negative selection of $V\beta6^{+}CD4^{+}$ cells. In view of the unexpected high frequency of "forbidden" $V\beta6^{+}$ lymphomas (two out of six definable cases), it seems that this interaction, which involves the CD3/TCR complex, MHC determinants, and the CD4 accessory molecule, may have resulted in stimulation rather than deletion. This is plausible because the same molecules are involved in intrathymic positive and negative selection. Such stimulatory signals may also be involved in the generation of the rather high proportion of biclonal lymphomas (two out of fourteen). In case 4, one member is indeed $V\beta6^{+}$.

Despite the presence of potentially autoreactive cells, there were no indications for autoimmune reactions in the animals. Several explanations can be given for this phenomenon. The lack of autoreactivity can be due to shortcomings in the functional properties of the $V\beta6^{+}$ lymphoma cells. Because the lymphoma cells most likely arose from an immature DP ($CD4^{+}CD8^{+}$) thymocyte, they have probably not been positively selected. Another possibility is that a loss of other essential membrane antigens, for example, those involved in cellular adhesion, underlies a diminished autoreactive capacity. It is also possible that the stimulatory $Mls\text{-}1^{a}$ antigens are not sufficiently expressed in the lymphomic lymphoid organs. Finally, clonal anergy to explain tolerance is seen in some (experimental) conditions (Rammensee et al., 1989; Blackman et al., 1990a). In this case, presumably autoreactive cells are blocked for their reactivity and hence do not mediate autoreactions and autoimmune symptoms. Clonal anergy is however not the most prominent way of naturally occurring tolerance to $Mls\text{-}1^{a}$ antigens (Rammensee et al., 1989; Blackman et al., 1990a).

In conclusion, our present findings suggest that lymphomagenesis in AKR mice occurs predominantly at the immature DP stage of T-cell development. This is followed by a defective clonal deletion in the lymphomic thymus and results in the generation of T cells with the phenotype of potentially autoreactive cells. These are the first data indicating a failure of the thymic deletion system under nonphysiological conditions.

### MATERIALS AND METHODS

### Mice

Male AKR/FuRdARij specific pathogen-free mice of 8–10 weeks old were obtained from the Central Animal Facility of the TNO Institute for Applied Radiobiology and Immunology, Rijswijk, The Netherlands. They were kept under clean conventional conditions. Spontaneous lymphomas were obtained from untreated mice at the age of 6 months or older.

### (Immuno-)histochemistry

At autopsy, thymus, spleen, and mesenteric lymph nodes were harvested and snap frozen in liquid nitrogen. For conventional light microscopy, 8-μm sections were stained with hematoxylin and eosin. For immunohistochemistry, 6-μm sections were fixed in acetone for 10 min at room temperature and then incubated with the first mAB. CD3 expression was detected using mAB YCD3-1 (Portoles et al., 1989) (a gift of Dr. K. Bottomly, Yale University School of Medicine, New Haven, CT). CD4 and CD8 antigens were determined using mAB MT4 (Pierres et al., 1984) and Lyt-2 (Ledbetter and Herzenberg, 1979), respectively (provided by Dr. G. Kraal, Free University, Amsterdam, The Netherlands). TCR Vβ usage was determined using mAB 44-22-1 (Vβ6 [Payne et al., 1988]; a gift of Dr. R. Schneider, University Hospital, Zurich, Switzerland), mAB KJ16 (Vβ8.1 and Vβ8.2 [Haskins et al., 1984]) and mAB KJ25 (Vβ3 [Pullen et al., 1988]), both provided by Drs. J. W. Kappler and P. Marrack, Howard Hughes Medical Institute, Denver, CO. The primary mABs were detected using an indirect method. YCD3-1, MT4, Lyt-2, 44-22-1, and KJ16 were used in combination with goat antirat IgG conjugated to horseradish peroxidase (Jackson Immunoresearch Laboratories, West Grove, PA) and rabbit antigoat Ig conjugated to horseradish peroxidase (Dakopatts, Glostrup, Denmark). Binding of KJ25 was detected using a combination of rabbit anti-hamster IgG conjugated to biotin, with subsequent detection by streptavidin-peroxidase (both from Jackson Immunoresearch Laboratories). Peroxidase activity was developed by 3,3-diaminobenzidine tetrahydrochlorid (DAB) with $H_2O_2$ as the substrate. The sections were counterstained with haematoxylin.

### Cytofluorographics

Cytofluorographic staining of thymocyte-cell suspensions from normal AKR mice was done using CD4 mAB GK1.5 (Dialynas et al., 1983) conjugated with biotin in combination with streptavidin-phycoerythrin. For CD8, mAB 53-6.7 (Becton Dickinson, Sunnyvale, CA) directly conjugated to FITC was used. Analysis of $10^6$ cells/mL was done using a FACScan (Becton Dickinson).

### Genomic DNA Analysis

Genomic DNA was isolated from cell suspensions by proteinase K digestion, precipitated with ethanol and redissolved in TRIS/EDTA, pH 8.0. Polymerase chain reaction (PCR) amplification was performed in a reaction buffer consisting of 50 mM KCl, 10 mM TRIS (pH 8.4), 4.0 mM $MgCl_2$, bovine serum albumin (100 $\mu$g/mL), each deoxyribonucleotide (dNTP) at 0.2 mM (Pharmacia, Uppsala, Sweden), 2 $\mu$g of AKR genomic spleen DNA, 100–300 ng of each of two oligonucleotide primers and 1 U of *Taq* DNA polymerase (Gibco/BRL, Bethesda, MD) in a total volume of 50 $\mu$L. The primers are based on known nucleotide sequences of murine T-cell receptor genes (Chien et al., 1984; Gascoigne et al., 1984; Patten et al., 1984; Chou et al., 1987): V$\beta$6: ACTGAAAACGATCTTCAAAA; V$\beta$8.1: GTCGCTGACAGCACGGAGAA; V$\beta$8.2: GCTCTTCTTCGTGCTCTCCAG, and a primer complementary to a conserved region of the J$\beta$ clusters: mJ$\beta$1: GAAACCAGGTCCGTGGTC. The reactions were performed for 35 cycles of denaturation (1 min at 94 °C), annealing (1 min at 60 °C), and extension (1 min at 72 °C). The PCR products were analyzed by agarose gel electrophoresis, and had a size, based on their location in the agarose gel, that was in agreement with the published sequences.

### ACKNOWLEDGMENTS

This work was supported in part by the Stichting Technische Wetenschappen (STW), project no. PCT99.2105.

*(Received May 14, 1991)*

*(Accepted September 30, 1991)*

### REFERENCES

Blackman M.A., Gerhard-Burgert H., Woodland D.L., Palmer E., Kappler J.W., and Marrack P. (1990a). A role for clonal inactivation in T cell tolerance to Mls-1ª. Nature **345:** 540–542.

Blackman M., Kappler J., and Marrack P. (1990b). The role of the T cell receptor in positive and negative selection of developing T cells. Science **248:** 1335–1341.

Chien Y., Gascoigne N.R.J., Kavaler J., Lee N.E., and Davis M.M. (1984). Somatic recombination in a murine T-cell receptor gene. Nature **309:** 322–326.

Chou H.S., Anderson S.J., Louie M.C., Godambe S.A., Pozzi M.R., Behlke M.A., Huppi K., and Loh D.Y. (1987). Tandem linkage and unusual RNA splicing of the T-cell receptor $\beta$-chain variable-region genes. Nature **84:** 1992–1997.

Dialynas D.P., Wilde D.B., Marrack P., Pierres A., Wall K.A., Havran W., Otten G., Loken M.R., Pierres M., Kappler J., and Fitch F.W. (1983). Characterization of the murine antigenic determinant, designated L3T4a, recognized by monoclonal antibody GK1.5: Expression of L3T4a by functional T cell clones appears to correlate primarily with class II MHC antigen-reactivity. Immunol. Rev. **74:** 29–56.

Frankel W.N., Rudy C., Coffin J.M., and Huber B.T. (1991). Linkage of Mls gene to endogenous mammary tumour viruses of inbred mice. Nature **349:** 526–528.

Gascoigne N.R.J., Chien Y., Becker D.M., Kavalar J., and Davis M.M. (1984). Genomic organization and sequence of T-cell receptor $\beta$-chain constant- and joining-region genes. Nature **310:** 387–391.

Happ M.P., Woodland D.L., and Palmer E. (1989). A third T-cell receptor $\beta$-chain variable region gene encodes reactivity to Mls-1ª gene products. Proc. Natl. Acad. Sci. USA **86,** 6293–6296.

Haskins K., Hannum C., White J., Roehm N., Kubo R., Kappler J., and Marrack P. (1984). The antigen specific, major histocompatibility complex-restricted receptor on T cells. VI. An antibody to a receptor allotype. J. Exp. Med. **160:** 452–471.

Kappler J.W., Staerz U., White J., and Marrack P. (1988). Self-tolerance eliminates T cells specific for Mls-modified products of the major histocompatibility complex. Nature **332:** 35–40.

Kim S.A., Evans L.H., Malik F.G., and Rouse R.V. (1991). Expression of retrovirus by thymic macrophages during AKR mouse leukemogenesis. *In:* Lymphatic tissues and in vivo immune responses: Proceedings of the 10th international conference on lymphatic tissues and germinal centres in immune reactions, Imhof B., Ed. (New York: Marcel Dekker). In press.

Ledbetter J.A., and Herzenberg L.A. (1979). Xenogeneic monoclonal antibodies to mouse lymphoid differentiation antigens. Immunol. Rev. **47:** 63–90.

Lee N.E., and Davis M.M. (1988). T cell receptor β-chain genes in BW5147 and other AKR tumors. Deletion order of murine Vβ gene segments and possible 5' regulatory regions. J. Immunol. **140:** 1665–1675.

MacDonald H.R., Hengartner H., and Pedrazzini T. (1988b). Intrathymic deletion of self-reactive cells prevented by neonatal anti-CD4 antibody treatment. Nature **335:** 174–176.

MacDonald H.R., Schneider R., Lees R.K., Howe R.C., Acha-Orbea H., Festenstein H., Zinkernagel R.M., and Hengartner H. (1988a). T-cell receptor Vβ use predicts reactivity and tolerance to Mls$^a$-encoded antigens. Nature **332:** 40–45.

Matsuzaki G., Yoshikai Y., Ogimoto M., Kishihara K., and Nomoto K. (1990). Clonal deletion of self-reactive T cells at the early stage of T cell development in thymus of radiation bone marrow chimeras. J. Immunol. **145:** 46–51.

Patten P., Yokota T., Rothbard J., Chien Y., Arai K., and Davis M. (1984). Structure, expression and divergence of T-cell receptor β-chain variable regions. Nature **312:** 40–46.

Payne J., Huber B.T., Cannon N.A., Schneider R., Schilhan M.W., Acha-Orbea H., MacDonald H.R., and Hengartner H. (1988). Two monoclonal rat antibodies with specificity for the β-chain variable region Vβ6 of the murine T-cell receptor. Proc. Natl. Acad. Sci. USA **85:** 7695–7698.

Pierres A., Naquet P., Van Agthoven A., Bekkhoucha F., Denizot F., Mishal Z., Schmitt-Verhulst A.-M., and Pierres M. (1984). A rat anti-mouse T4 monoclonal antibody (H129.19) inhibits the proliferation of Ia-reactive T cell clones and delineates two phenotypically distinct (T4$^+$,Lyt-2,3$^-$, and T4$^-$,Lyt-2,3$^+$) subsets among anti-Ia cytolytic T cell clones. J. Immunol. **132:** 2775–2782.

Portoles P., Rojo J., Golby A., Bonneville M., Gromkowski S., Greenbaum L., Janeway G.A., Murphy D.B., and Bottomly K. (1989). Monoclonal antibodies to murine CD3ε define distinct epitopes, one of which may interact with CD4 during T cell activation. J. Immunol. **142:** 4169–4175.

Pullen A.M., Marrack P., and Kappler J.W. (1988). The T-cell repertoire is heavily influenced by tolerance to polymorphic self-antigens. Nature **335:** 796–801.

Rammensee H.-G., Kroschewski R., and Frangoulis B. (1989). Clonal anergy induced in mature Vβ6$^+$ T lymphocytes on immunizing Mls-1$^b$ mice with Mls-1$^a$ expressing cells. Nature **339:** 541–544.

Ramsdell F., and Fowlkes B.J. (1990). Clonal deletion versus Clonal anergy: The role of the thymus in inducing self tolerance. Science **248:** 1342–1348.

Richie E.R., McEntire B., Crispe N., Kimura J., Lanier L.L., and Allison J.P. (1988). α/β T-cell antigen receptor gene and protein expression occurs at early stages of thymocyte differentiation. Proc. Natl. Acad. Sci. USA **85:** 1174–1178.

Schuurman H.-J., Henzen-Logmans S.C., and Kluin P.M. (1992). Non-Hodgkin's lymphomas. *In:* Clinical Cancer Markers: Diagnosis, prognosis and monitoring, Sell, S. Ed. Clifton, NJ: The Human Press. In press.

Tempelis L.D. (1987). Thymic epithelium controls thymocyte expression of preleukemic phenotype and leukemogenic retroviruses. J. Immunol. **138:** 3555–3565.

Webb S.R., and Sprent J. (1990). Response of mature unprimed CD8$^+$ T cells to Mls$^a$ determinants. J. Exp. Med. **171:** 953–958.

